# Omicron variant Spike-specific antibody binding and Fc activity is preserved in recipients of mRNA or inactivated COVID-19 vaccines

**DOI:** 10.1126/scitranslmed.abn9243

**Published:** 2022-03-15

**Authors:** Yannic C. Bartsch, Xin Tong, Jaewon Kang, María José Avendaño, Eileen F. Serrano, Tamara García-Salum, Catalina Pardo-Roa, Arnoldo Riquelme, Yongfei Cai, Isabella Renzi, Guillaume Stewart-Jones, Bing Chen, Rafael A. Medina, Galit Alter

**Affiliations:** ^1^ Ragon Institute of MGH, MIT, and Harvard; Cambridge, MA 02138, USA; ^2^ Department of Pediatric Infectious Diseases and Immunology, School of Medicine, Pontificia Universidad Católica de Chile, Santiago 8331010, Chile; ^3^ Advanced Interdisciplinary Rehabilitation Register (AIRR) – COVID-19 Working Group, Faculty of Medicine, Pontificia Universidad Católica de Chile, Santiago 8331010, Chile; ^4^ Department of Gastroenterology, School of Medicine, Pontificia Universidad Católica de Chile, Santiago 8331010, Chile; ^5^ Department of Health Sciences, Faculty of Medicine, Pontificia Universidad Católica de Chile, Santiago 8331010, Chile; ^6^ Division of Molecular Medicine, Boston Children's Hospital, Harvard Medical School, Boston, MA 02115, USA; ^7^ Moderna Inc., Cambridge, MA 02138, USA; ^8^ Department of Microbiology, Icahn School of Medicine at Mount Sinai, New York, NY 10029, USA

## Abstract

The Omicron variant of SARS-CoV-2 has been shown to evade neutralizing antibodies elicited by vaccination or prior infection. Despite the dramatic global spread of the Omicron variant, even among highly vaccinated populations, death rates have not increased concomitantly. These data suggest that immune mechanisms beyond antibody-mediated virus neutralization may protect against severe disease. In addition to neutralizing pathogens, antibodies contribute to control and clearance of infections through Fc-effector mechanisms. Here we probed the ability of vaccine-induced antibodies to drive Fc-effector activity against the Omicron variant using samples from individuals receiving one of three SARS-CoV-2 vaccines. Despite a substantial loss of IgM, IgA, and IgG binding to the Omicron variant Receptor Binding Domain (RBD) in samples from individuals receiving BNT162b2, mRNA-1273, and CoronaVac vaccines, stable binding was maintained against the full-length Omicron Spike protein. Compromised RBD binding IgG was accompanied by a loss of cross RBD-specific antibody Fcγ receptor (FcγR) binding in samples from individuals who received the CoronaVac vaccine, but RBD-specific FcγR2a and FcγR3a binding was preserved in recipients of mRNA vaccines. Conversely, Spike protein-specific antibodies exhibited persistent but reduced binding to FcγRs across all three vaccines, though higher binding was observed in samples from recipients of mRNA vaccines. This was associated with preservation of FcγR2a and FcγR3a binding antibodies and maintenance of Spike protein-specific antibody-dependent natural killer cell activating antibodies. Thus, despite the loss of Omicron neutralization, vaccine-induced Spike protein-specific antibodies continue to drive Fc-effector functions, suggesting a capacity for extra-neutralizing antibodies to contribute to disease control.

## Teaser

SARS-CoV-2-specific antibodies induced by mRNA or inactivated virus vaccines maintain Omicron variant-specific Fc-mediated effector mechanisms.

## INTRODUCTION

Antibodies represent the primary correlate of immunity following immunization with nearly all licensed vaccines (*1*), providing protection either through direct blockade of infection or through their ability to leverage the immune system to eliminate pathogens, should the pathogens breach the portal of entry (*2*). Emerging data from severe acute respiratory syndrome coronavirus 2 (SARS-CoV-2) Phase 3 vaccine studies clearly demonstrate a critical association between neutralizing and binding antibodies and protection against severe coronavirus disease 2019 (COVID-19) (*3*). Yet, the emergence of SARS-CoV-2 variants of concern (VOC), including the Omicron variant that evades neutralizing antibodies, has led to increased breakthrough infections globally among vaccinated individuals. Thus far, despite this striking rise in breakthrough infections, a concomitant rise in severe disease and death has not been observed. Unlike previous VOCs, emerging data suggest that Omicron exhibits reduced angiotensin converting enzyme 2 (ACE2) binding (*4*), and is largely an upper respiratory disease (*5*, *6*). However, whether this compartmentalization of disease is related to differential viral infectivity alone or also related to persistent vaccine-induced immunity remains incompletely understood. The lower hospitalization rates among vaccinated individuals (*7*) suggest that vaccine-mediated protection may still persist despite loss of neutralizing antibody activity. Although it seems that transmission blockade may be lost against Omicron, disease attenuation may still be maintained through alternative vaccine-induced immune responses that critically modulate disease severity, which is the ultimate goal of vaccination.

Beyond blockade of infection, cellular immune responses can directly or indirectly contribute to protection against severe disease. T cells may directly recognize and eliminate infected cells (*8*). In addition, binding antibodies with the capability of interacting with Fc receptors (FcRs), found on immune cells, can leverage the antiviral activity of the innate immune system (*9*). These binding antibodies can rapidly drive opsonophagocytic clearance, promote killing of infected cells, and elicit the release of pro- or anti-inflammatory mediators. Each of these features have been linked to protection against several viruses, including Influenza (*10*, *11*), Ebola virus (*12*, *13*), and HIV (*14*). Previous studies have shown that attenuated or delayed SARS-CoV-2 IgG responses with compromised Fcɣ receptor (FcɣR) binding have been linked to fatal SARS-CoV-2 infection (*15*). Moreover, patients hospitalized with COVID-19 are less likely to have opsonophagocytic antibodies compared to non-hospitalized patients (*16*). Furthermore, several SARS-CoV-2 neutralizing monoclonal antibodies require Fc effector functions to confer protection against SARS-CoV-2 infection and disease in animal models, collectively highlighting the importance of extra-neutralizing Fc effector functions in immunity to SARS-CoV-2 (*17*–*20*). However, whether Fc activity persists to provide protection against Omicron remains unclear. Here we show diminished antibody isotype binding to the Omicron receptor binding domain (RBD) across vaccine platforms, but persistent, albeit reduced, Fc-activity to the Omicron Spike protein. This likely contributes to rapid control and clearance of viral infection, thereby attenuating disease severity.

## RESULTS

### Omicron RBD recognition was reduced in samples from individuals vaccinated with BNT162b2, mRNA-1273, and CoronaVac.

**Fig. 1.**
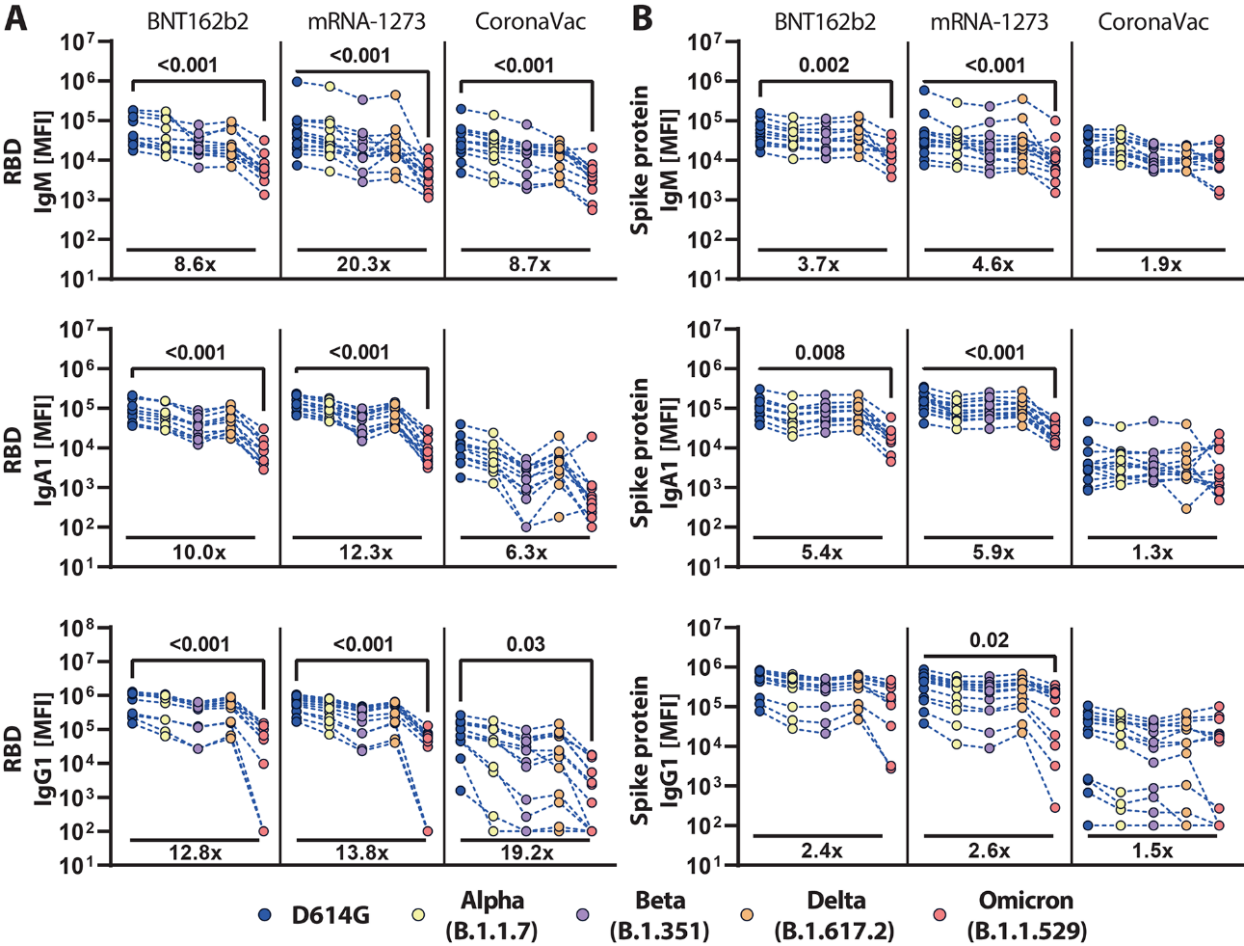
Vaccine-induced antibody binding to the Spike protein of SARS-CoV-2 is maintained across variants of concern. Individuals received the full dose regimen of the BNT162b2 mRNA vaccine (n = 11), mRNA-1273 vaccine (n=14), or the aluminum-adjuvanted inactivated particle vaccine CoronaVac (n=13). Samples were obtained from vaccine recipients 13 to 19 days after the second dose. IgM, IgA1, and IgG1 binding titers to D614G, Alpha (B.1.1.7), Beta (B.1.351), Delta (B.1.617.2), and Omicron (B.1.1.529) VOC (**A**) RBDs or (**B**) full-length Spike protein were measured by a Luminex assay. The average value of technical replicates is shown. The data was corrected for background and negative values were set to 100 for graphing purposes. A two-sided Kruskal-Wallis test with a Benjamini-Hochberg post-test correcting for multiple comparisons was used to test for statistical differences between D614G and Omicron titers. P-values for significantly different features (p≤0.05) are shown; fold-change reduction of omicron titers compared to D614G are shown below each dataset. MFI, median fluorescence intensity.

Despite the substantial loss of vaccine-induced neutralization against the Omicron VOC, persistence of vaccine-induced antibody binding may continue to confer protection against disease through extra-neutralizing antibody functions that have been linked to natural resolution of infection and vaccination (*9*, *15*, *17*). Thus, we probed the persistence of vaccine-induced antibody binding to recombinant RBD across SARS-CoV_2 VOCs (**Fig. 1A**). Persistence of RBD recognition was compared using plasma samples from recipients of 3 vaccine platforms, including the Moderna mRNA-1273 (*21*), Pfizer/BioNtech BNT162b2 (*22*), and Sinovac CoronaVac (*23*). All samples were collected 13 to 19 days after the second vaccine dose.

Comparable IgM responses were observed across all vaccine platforms to the RBD from D614G, Alpha (B.1.1.7), Beta (B.1.351), and Delta (B.1.617.2), with a significant (p<0.001) loss of Omicron (B.1.1.529) recognition by all three platforms (**Fig. 1A**). mRNA vaccines induced stronger IgA RBD-specific cross-reactive responses, that were compromised, but not completely lost, for Omicron. IgA responses were observed across RBD VOCs in samples from those receiving CoronaVac vaccination. Robust IgG responses were observed for mRNA vaccines that were relatively stable across VOC RBDs; this included a decrease in, but not complete loss of, recognition of the Omicron RBD. As expected, CoronaVac induced lower IgG responses across VOC RBDs and also exhibited diminished binding to the Omicron RBD. These data point to a persistent, albeit lower, recognition of the Omicron RBD across isotypes by these 3 vaccine platforms.

### Recognition of Omicron Spike protein was maintained across vaccine platforms.

Although most neutralizing antibodies target the Spike antigen on or proximal to the RBD (*24*), Fc-functional antibodies that drive viral clearance or killing of infected cells can target the whole surface of the Spike antigen. Thus, we next profiled isotype recognition across Spike proteins from VOCs (**Fig. 1B**). All vaccines induced Spike protein-specific IgM responses across most VOCs and exhibited attenuated binding to the Omicron Spike protein. IgA responses that reacted against Spike protein VOCs were most robustly induced by the BNT162b2 and mRNA-1273 vaccines, but these samples still exhibited a partial decline in recognition of the Omicron Spike protein. Conversely, the CoronaVac vaccine elicited lower IgA responses across Spike proteins from VOCs that were completely preserved against Omicron. Moreover, BNT162b2 and mRNA-1273 mRNA vaccines induced the highest degree of Spike protein IgG binding against all measured VOCs; only a moderate loss of recognition of the Omicron Spike protein was observed. Likewise, no difference in IgG binding to the Omicron N-terminal domain (NTD) compared to D614G NTD in BNT162b2 vaccinated individuals was observed (**fig. S1**). Interestingly, despite the lower overall IgG titers induced by the CoronaVac vaccine, IgG responses recognized the Omicron Spike proteins similarly to the D614G spike protein, pointing to robust preservation of Spike protein-specific IgG immunity across the 3 vaccine platforms.

### Omicron-specific FcγR binding activity was variable across vaccine platforms.

**Fig. 2.**
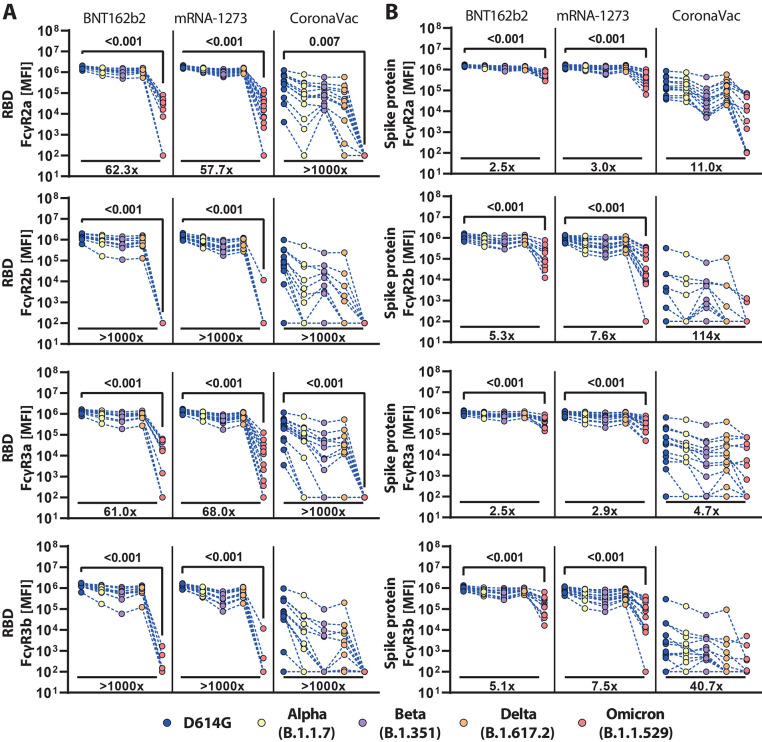
FcγR binding antibody profiles against SARS-CoV-2 VOCs vary across vaccine type. Individuals received the full dose regimen of the BNT162b2 mRNA vaccine (n = 11), mRNA-1273 vaccine (n=14), or the aluminum-adjuvanted inactivated particle vaccine CoronaVac (n=13). Samples were profiled 13 to 19 days after the last vaccine dose. Binding to FcγR2a, FcγR2b, FcγR3a, and FcγR3b of antibodies specific to the (**A**) RBD or (**B**) full-length spike protein of D614G, Alpha (B.1.1.7), Beta (B.1.351), Delta (B.1.617.2), or Omicron (B.1.1.529) were determined by a Luminex assay. The average value of technical replicates is shown. The data was corrected for background and negative values were set to 100 for graphing purposes. A two-sided Kruskal-Wallis test with a Benjamini-Hochberg post-test correcting for multiple comparisons was used to test for statistical differences between D614G-specific and Omicron-specific antibody activity. P-values for significant different features (p≤0.05) are shown; fold-change reduction of omicron binding activity compared to D614G is shown below each dataset.

The ability of antibodies to leverage Fc-effector functions depends on their ability to interact with FcRs found on all immune cells (*25*). Thus, we profiled the ability of vaccine-induced RBD and Spike protein-specific antibodies to interact with the four low affinity FcγRs found in humans, known to regulate and drive antibody effector functions (*26*). mRNA vaccines induced robust cross-reactive RBD-specific FcγR binding antibodies, but exhibited a near complete loss of inhibitory FcγR2b and neutrophil-specific FcγR3b binding in response to the Omicron variant RBD; in contrast, opsonophagocytic FcγR2a and cytotoxic FcγR3a binding to the Omicron variant RBD remained detectable (**Fig. 2A**). Omicron FcR binding was reduced but detected more robustly for Spike protein-specific antibodies (**Fig. 2B**). CoronaVac induced an intermediate degree of RBD-specific FcγR binding antibodies across VOCs but exhibited a near complete loss of Omicron RBD-specific FcγR binding, despite the ability to bind to RBD (**Fig. 1A** **,** **Fig. 2A**). These data point to qualitative differences in antibody Fc-binding capabilities that are not always linked to antibody titers. Although CoronaVac induced comparable concentrations of D614G and Omicron Spike protein-specific IgG antibodies (**Fig. 1B**), Spike protein-specific antibodies elicited by CoronaVac exhibited a more profound decline in Omicron-specific FcγR-binding (**Fig. 2B**). Of note, these patterns were sex-independent (**fig. S2**). However, a common pattern of Omicron Spike protein-specific FcγR-binding loss was observed across the three platforms, marked by a selective persistence of higher opsonophagocytic FcγR2a and cytotoxic FcγR3a binding, and a sharper decline of inhibitory FcγR2b and neutrophil-activating FcγR3b binding.

### Disproportionate loss of Omicron RBD-specific, but not Spike protein-specific, antibody Fc binding

**Fig. 3.**
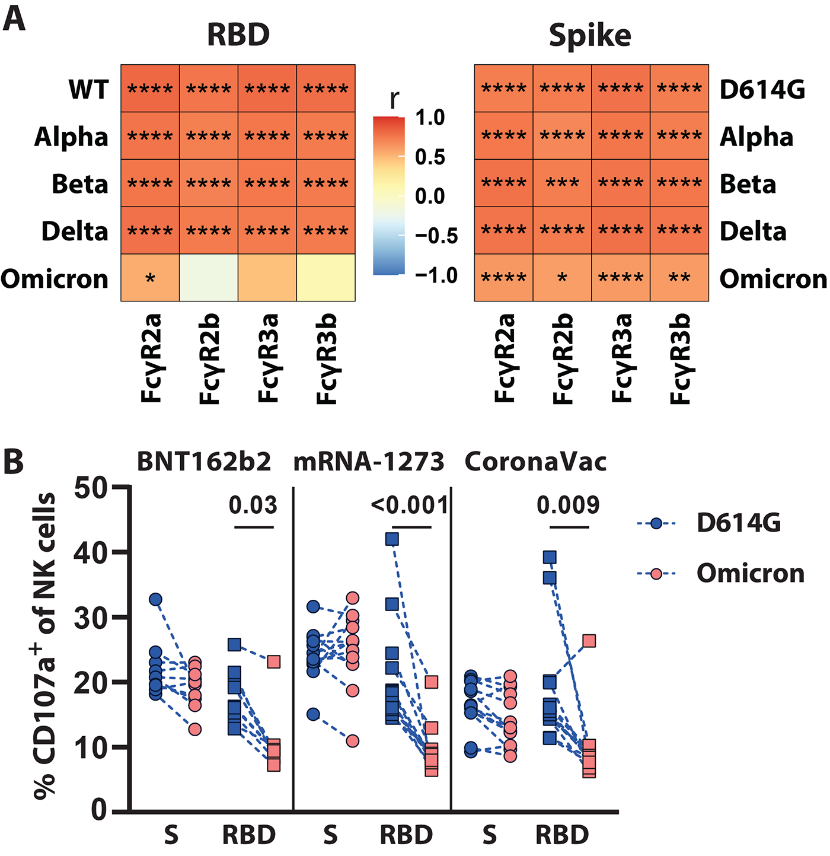
Vaccine induced binding titers correlate with FcγR binding across VOCs. The heatmaps show the Spearman correlation between the D614G or VOC (**A**) RBD-specific or (**B**) Spike protein-specific IgG1 titers and antibody binding to the respective FcγRs. The color indicates the correlation coefficient (r) as indicated by the color key. Asterisks indicate statistically significant correlations; *p≤0.05, **p≤0.01, ***p≤0.001, ****p≤0.0001.

To begin to gain a deeper understanding in the relationship of Omicron-specific antibody binding and FcγR-driven biology, we constructed correlation matrices for IgG1 binding titers and individual FcγR binding activity for the different VOCs. As expected, significant (p<0.05), positive (r>0.7) associations were noted between RBD- or Spike protein-specific antibody-binding concentrations and FcγR binding activity across the D614G, Alpha, Beta, and Delta variants (**Fig. 3A and B**). The relationship between Spike protein-specific antibody binding titers and FcγR2b was slightly less robust, pointing to some variation in the ability of vaccine-induced antibodies to retain and leverage this inhibitory human FcR against the Beta VOC. Strikingly, lower correlations were noted in Omicron RBD-specific binding antibody concentrations and all FcγR binding activity. RBD-specific antibodies were solely significantly (p<0.05) correlated with FcγR2a binding activity. Conversely, Omicron Spike protein-specific antibody binding concentrations continued to correlate with FcγR binding activity, albeit weaker than relationships previously observed for other VOCs. Moreover, the relationship between Omicron Spike protein-specific binding and FcγR binding was maintained more robustly for FcγR2a and FcγR3a, potentially, marking the retention of particular antibody effector functions.

**Fig. 4.**
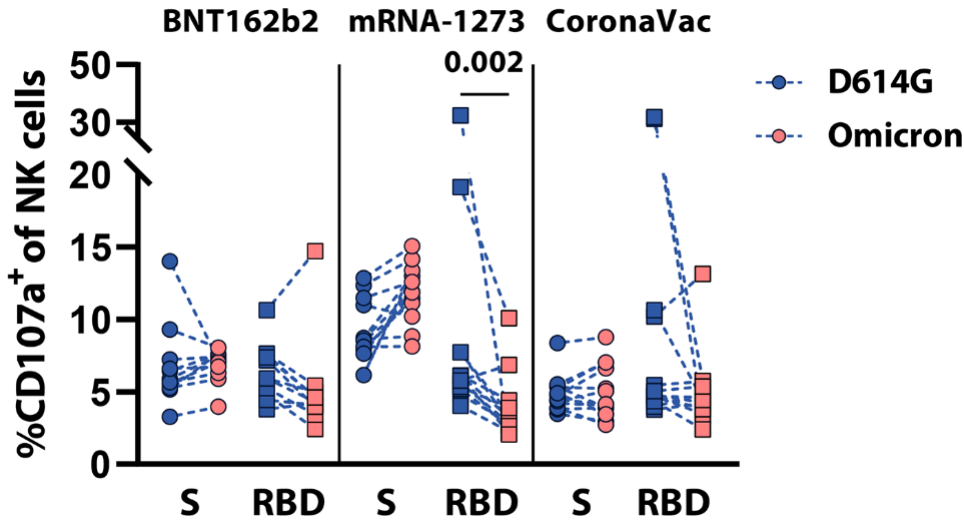
Spike protein-specific, but not RBD-specific, Fc-mediated effector activity is preserved in samples from vaccinated individuals. Antibody-dependent NK cell activation (ADNKA) was assessed by CD107a expression, a marker of NK cell degranulation, on CD3^-^CD56^+^ NK cells. Samples were incubated with D614G (blue) or Omicron (red) Spike (S) protein (circle) or RBD (square) and samples from recipients of 1 of 3 vaccine platforms: BNT162b2 (n=11), mRNA-1273 (n=13), and CoronaVac (n=13). The assay was performed with three different NK cell donors and the average value of all donors is shown. A two-sided Kruskal-Wallis test with a Benjamini-Hochberg post-test correcting for multiple comparisons was used to test for statistical differences between D614G and omicron. Significance was defined as p≤0.05 and only p-values for significantly different comparisons are shown.

To further investigate whether Fc-effector was maintained against Omicron, an antibody-dependent natural killer (NK) cell activation (ADNKA) assay, which mainly relies on FcγR3a binding, was performed (**Fig. 4** **and fig. S3**). As expected, Spike protein-specific antibodies to the D614G and Omicron Spike protein induced comparable degrees of NK cell activation (marked by the up-regulation of CD107a, a marker of NK cell degranulation) across the three vaccine platforms (*27*). Conversely, only antibodies to the D614G RBD but not Omicron were able to robustly activate NK cells. Moreover, as expected, RBD-specific antibody-mediated NK cell degranulation declined across the 3 vaccine platforms to the Omicron-RBD; however, this decrease was the most apparent in Omicron Spike protein-specific antibodies isolated from mRNA-1273 recipients. Thus, these data highlight the robust preservation of Omicron Spike protein-specific Fc-effector functions, despite the loss of RBD-specific antibody activity.

## DISCUSSION

As SARS-CoV-2 continues to evolve, the virus has acquired a progressive collection of mutations preferentially located within the S1 domain of the Spike antigen, within or proximal to the RBD (*28*). Because many of the most potent neutralizing antibodies bind to the RBD, aimed at interfering or blocking interactions with ACE2, both vaccine-induced neutralizing antibodies and monoclonal therapeutics have progressively lost neutralization potency against emerging VOCs (*29*, *30*). Yet unlike previous VOCs, the Omicron variant accumulated more than 40 mutations, including 30 in the Spike protein, that, to date, represents the most profound escape from natural and vaccine-induced neutralizing antibody activity. This loss of neutralization, coupled to enhanced ACE2-binding, accounts for the remarkable rise in transmission events globally. However, as a second line defense following infection, both direct and indirect cellular mechanisms contribute to pathogen control and clearance. Specifically, T cells may directly recognize and kill infected cells (*8*). Additionally, antibodies able to leverage innate immune activity can both drive the rapid elimination of viral particles as well as deploy the cytotoxic power of the immune system to kill infected cells (*25*). Although emerging data point to persistent COVID-19 vaccine-induced T cell recognition of Omicron (*31*), it was unclear whether vaccine-induced antibodies continue to leverage Fc-activity against the Omicron VOC.

Here we observed a more pronounced loss of antibodies targeting the Omicron RBD compared to Omicron Spike protein and compared to FcR binding across vaccine platforms, likely linked to the preferential incorporation of mutations in the S1 domain of the SARS-CoV-2 Spike protein. However, unlike neutralizing antibodies that must target a limited number of regions on the Spike protein involved in attachment, positioning of the RBD, or fusion, antibodies that mediate Fc-activity can likely bind across the entire antigenic surface. Likewise, we did not observe a reduction in IgG1 binding titers to the Omicron N-terminal domain (NTD) compared to the D614G variant. Fc-activity solely requires formation of immune complexes and arrangements of antibodies with Fc-domains that are accessible to local immune cells. The reduced but persistent binding of IgG antibodies to the Omicron Spike antigen across the mRNA and inactivated vaccine platforms suggests that vaccine-induced antibodies may continue to opsonize both virus and virally infected cells. Thus, although neutralizing antibodies are likely to be key to blocking transmission, non-neutralizing antibodies able to leverage Fc-biology, including NK cell-mediated cytotoxicity, may contribute to the elimination of infected cells soon after infection has occurred.

Although the three vaccines maintained robust binding to Omicron Spike protein-specific FcγR2a, a selective loss of FcγR2b and FcγR3b was observed across the platforms. FcγR2b is the sole low-affinity inhibitory receptor in humans, likely involved in attenuating inflammatory activity (*26*). Likewise, FcγR3b is solely expressed on neutrophils, likely critical for rapid opsonophagocytic clearance of opsonized viral particles (*26*). Whereas continued binding to FcγR2a and FcγR3a may lead to continued clearance of particles and killing of infected cells, the loss of FcγR2b and FcγR3b may result in a more inflammatory response that may attenuate severity and death. Likewise, emerging epidemiological reports suggest that Omicron infection is less severe and causes mild to moderate symptomatic infection (*32*, *33*). However, real-world comparisons of symptom severity across vaccine platforms are needed to provide enhanced resolution of the roles of individuals FcγRs in attenuating disease.

Our study has limitations. We compared humoral responses across only three vaccine platforms on a limited set of samples. Recipients received a two-dose regimen and samples were analyzed within 4 weeks after vaccination. Thus, it remains unclear how durable the observed VOC responses are. Furthermore, the effects of a third dose in might further increase VOC-specific responses. Thus, future studies across additional platforms and in larger cohorts, including those with breakthrough infections, are needed. Studies investigating booster doses may also provide insights into the precise antibody functions that may be key to attenuating COVID-19.

Although many developed countries have begun aggressive boosting campaigns, the majority of the world remains incompletely vaccinated. Thus, understanding the ability of distinct vaccines to confer immunity against Omicron is urgently needed as efforts are made to increase global vaccine access. Moreover, defining the immunological mechanisms that contribute to disease attenuation in the absence of neutralization may provide key insights to guide effective pan-variant SARS-CoV-2 vaccine design and boosting campaigns. Here we demonstrate the persistence of Omicron Spike protein-binding antibodies, but reduced RBD-specific binding antibodies and varying Fc-activating potential across vaccine platforms. These data provide initial insights on persisting mechanisms that may contribute to disease attenuation despite the loss of neutralization to the Omicron VOC.

## MATERIALS AND METHODS

### Study Design

To compare antibody responses elicited by the different vaccines, samples were obtained from individuals who were vaccinated with full dose regimens as recommended by the respective manufacturer. As part of a phase 1 clinical trial in the US (NCT04283461) individuals (20 to 53 years old; median: 30 years old; 50% female) received two doses of 100 μg mRNA-1273 at days 0 and 28. Plasma samples taken two weeks after the second dose (*34*). BNT162b2 vaccinated individuals received 30 μg BNT162b2 (23 to 53 years old; median: 36 years old; 81% female) at days 0 and 21 and plasma samples were taken 13 to 19 days after the second dose. Individuals (23 to 46 years old; median: 31 years old; 61% female) received two doses of 600U CoronaVac four weeks apart and plasma samples were taken 13 to 19 days after the second dose (*35*). No immunocompromising comorbidities were reported, and we did not observe a sex bias in our dataset (**fig. S2**). No data points were omitted from the analysis. For the BNT162b2 and CoronaVac study, informed written consent was obtained under protocol 200829003 which was reviewed and approved by the Scientific Ethics Committee at Pontificia Universidad Católica de Chile (PUC). This study was overseen and approved by the Mass General Institutional Review Board (IRB #2020P00955 and #2021P002628). Buffy coats from healthy volunteers were collected and processed by the Massachusetts General Hospital (MGH) Blood Bank. All donors were 18 years of age or older and gave signed consent. Samples were deidentified before use and the study was approved by the MGH Institutional Review Board.

### Antigens

Full-length Spike and RBD (Arg319-Phe541 of the Spike protein) antigens for the D614G, alpha (B.1.1.7), beta (B.1.351), and delta (B.1.617.2) VOCs were obtained from Sino-Biologicals. Omicron (B.1.1.529) RBD (Arg319-Phe541 of the Spike protein) and N-terminal domain (NTD; Met1-Asp287) antigens were generously provided by Moderna Inc. Omicron Spike protein was produced in-house in HEK293F cells (Thermo Fisher Scientific; Catalog number: A14527) as described before (*36*). All RBD and NTD antigens as well as stabilized (hexa-pro) Spike protein of D614G or respective variants were produced in mammalian HEK293 cells.

### IgG subclass, isotype, and FcγR binding

Antigen-specific antibody subclasses and isotypes, as well as FcγR binding, were analyzed by Luminex multiplexing in technical replicates. The antigens were coupled to magnetic Luminex beads (Luminex Corp) by carbodiimide- N-hydroxysulfosuccinimide (Sulfo-NHS) ester-coupling with an individual region per antigen. Coupled beads were incubated with different plasma dilutions (1:100 for IgG2, IgG3, IgG4, IgM, and IgA1; 1:500 for IgG1 and 1:1,000 for FcγR probing) for 2 hours at room temperature in 384 well plates (Greiner Bio-One). Unbound antibodies were removed by washing and subclasses and isotypes were detected with a respective phycoerythrin (PE)-conjugated antibody (anti-human IgG1 (Cat# 9052-09, RRID:AB_2796621), IgG2 (Cat# 9060-09, RRID:AB_2796635), IgG3 (Cat# 9210-09, RRID:AB_2796701), IgG4 (Cat# 9200-09, RRID:AB_2796693), IgM (Cat# 9020-09, RRID:AB_2796577), or IgA1 (Cat# 9130-09, RRID:AB_2796656) all from Southern Biotech) at a 1:100 dilution. For analysis of FcγR binding PE-Streptavidin (Agilent Technologies) was coupled to recombinant and biotinylated human FcγR2a, FcγR2b, FcγR3a, or FcγR3b protein. Coupled FcγRs were used as a secondary probe at a 1:1000 dilution. After a 1 hour incubation, excessive secondary reagent was removed by washing and the relative antibody concentration per antigen was determined on an IQue analyzer (IntelliCyt).

### Antibody-Dependent-NK-Activation (ADNKA)

For the ADNKA assay, MaxiSorp enzyme-linked immunosorbent assay (ELISA) plates (Thermo Fisher Scientific) were coated with respective antigens for 2 hours at room temperature and then blocked with 5% bovine serum albumin (Sigma-Aldrich). Fifty μl of 1:50 diluted plasma samples were added to the wells and incubated overnight at 4°C. NK cells were isolated from buffy coats from healthy donors (as mentioned above) using the RosetteSep NK cell enrichment kit (STEMCELL Technologies) and stimulated with recombinant human interleukin (IL)-15 (1ng/ml, STEMCELL Technologies) at 37°C overnight. NK cells were added to the washed ELISA plates and incubated together with 1μg/ml anti-human CD107a brilliant violet (BV) 605 (BioLegend Cat# 328634, RRID:AB_2563851), brefeldin A (Sigma-Aldrich), and monensin (BD Biosciences) for 5 hours at 37°C. Next, cells were surface stained with 62.5 ng/ml CD56 phycoerythrin (PE)-Cy7 (BD Biosciences Cat# 335791, RRID:AB_399970) and 0.25 μg/ml CD3 allophycocyanin (APC)-Cy7 (BioLegend Cat# 300426, RRID:AB_830755). After fixation and permeabilization with FIX & PERM Cell Permeabilization Kit (Thermo Fisher Scientific), cells were stained for intracellular macrophage inflammatory protein (MIP)-1β BV421 (BD Biosciences Cat# 562900, RRID:AB_2737877, 0.25 μl per well). NK cells were defined as CD3^-^CD56^+^ single lymphocytes and frequencies of CD107a^+^ or MIP1β^+^ NK cells were determined using an iQue analyzer (Intellicyt). The ADNKA analysis was performed with NK cells from three different donors (biological replicates) and the average of the three donors is shown.

### Statistical analysis

Raw, individual-level data are presented in data file S1. If not stated otherwise, non-normal distributions were assumed. Plots were generated and statistical differences between two groups were calculated in Graph Pad Prism V.8. A Kruskal-Wallis test with a Benjamini-Hochberg post-test correcting for multiple comparisons was used to test for statistical differences between D614G-specific and omicron-specific titers.

## Supplementary Materials

### Other Supplementary Material for this manuscript includes the following:

MDAR Reproducibility Checklist
