# Supplementary material for: Omicron variant Spike-specific antibody binding and Fc activity is preserved in recipients of mRNA or inactivated COVID-19 vaccines
Source: Sci Transl Med. 2022 Mar 15:eabn9243. doi: 10.1126/scitranslmed.abn9243 (PMC8995028; doi:10.1126/scitranslmed.abn9243)
Supplement: Supplementary file 1 — Figs. S1 to S3 [file scitranslmed.abn9243_sm.pdf]

Science Translational Medicine
AAAS

Supplementary Materials for

Omicron variant Spike-specific antibody binding and Fc activity is preserved in recipients of mRNA or inactivated COVID-19 vaccines

Yannic C. Bartsch *et al.*

Corresponding authors: Galit Alter, galter@mgh.harvard.edu; Rafael A. Medina, rmedina@uc.cl

DOI: 10.1126/scitranslmed.abn9243

The PDF file includes:

Figs. S1 to S3

Other Supplementary Material for this manuscript includes the following:

MDAR Reproducibility Checklist
Data file S1

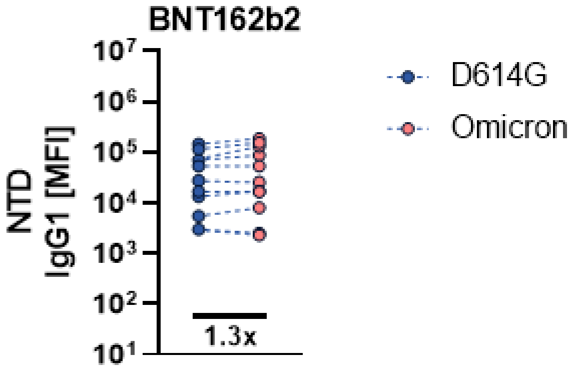

Fig. S1: IgG1 binding titers to NTD of D614G or Omicron are indistinguishable in BNT162b2 vaccinated individuals. IgG1 binding titer to the D614G or Omicron N-terminal domain (NTD) in BNT162b2 vaccinated individuals (n=11) was determined by Luminex. Fold change between the two variants is shown below the dataset. MFI, median fluorescence intensity.

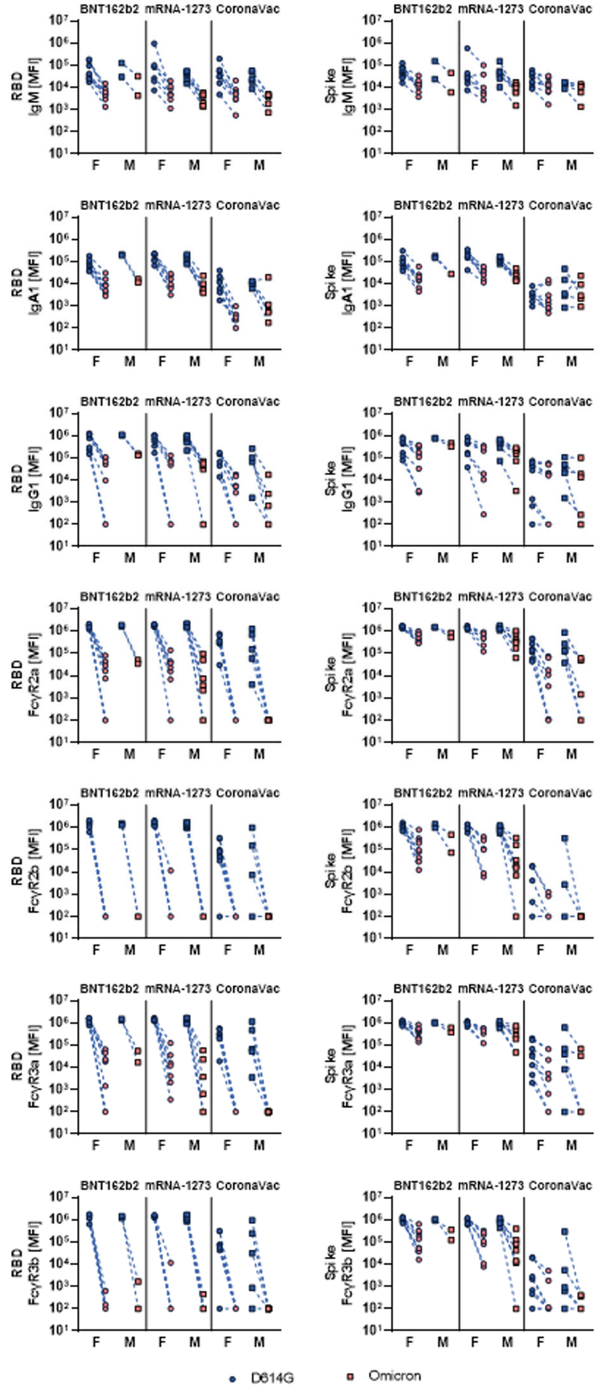

Fig. S2: Humoral immune responses were stratified by sex. Females (F, circles) and males (M, squares) received full dose regimens of the BNT162b2(n_{female}=9, n_{male}=2), mRNA-1273 (n_{female}=7, n_{male}=7), or CoronaVac (n_{female}=8, n_{male}=5) vaccines. Samples were taken 13 to 19 days after the second dose. (**A and B**) IgM, IgA1, and IgG1 binding titers against the RBD (A) or full-length spike protein (B) of D614G/WT (blue) or the omicron variant (red) are shown. (**C and D**) Binding to FcγR2a, FcγR2b, FcγR3a, and FcγR3b to D614G or the Omicron variant RBD (C) or full-length spike protein (D) were measured by Luminex. Background corrected data is shown and negative values were set to 100 for graphing purposes.

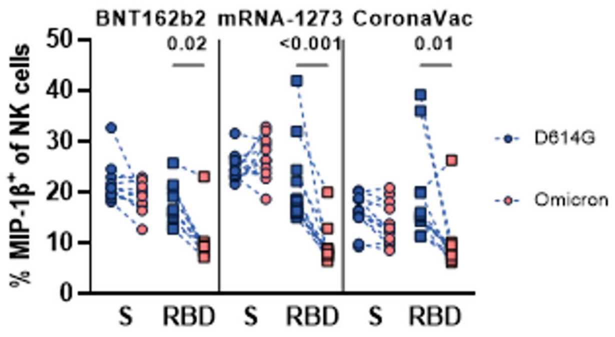

Fig. S3: Omicron Spike protein-specific, but not RBD-specific, antibodies maintain the ability to induce MIP-1β expression in NK cells. Antibody-dependent natural killer (NK) cell activation (ADNKA) against the D614G (blue) or Omicron (red) spike protein (S, square) or receptor binding domain (RBD, circle) was assessed by macrophage inflammatory protein (MIP)-1β expression in $CD3^{-}CD56^{+}$ NK cells across the 3 vaccine platforms: BNT162b2 (n=11), mRNA-1273 (n=13), and CoronaVac (n=13). The assay was performed with three different NK cell donors and the average value of all donors is shown. A Kruskal-Wallis test with a Benjamini-Hochberg post-test correcting for multiple comparisons was used to test for statistical differences between D614G and Omicron. Significance was defined as $p \leq 0.05$ and only p-values for significant different comparisons are shown.
